# One-Pot Synthesis of PtBi-Co_X_ Alloys for Electrochemical Nitrate Reduction to Ammonia

**DOI:** 10.3390/ma19101953

**Published:** 2026-05-09

**Authors:** Yingfei Liu, Yuxuan Wang, Xiyuan Sun, Chong Peng, Zhe Pang, Dafu Zhao, Kefeiyang Hu, Jiaqian Que, Xingbo Huang, Yong Liu

**Affiliations:** 1 State Key Laboratory of Advanced Technology for Materials Synthesis and Processing, School of Materials Science and Engineering, Wuhan University of Technology, Wuhan 430070, China; liu714961@163.com (Y.L.); 18952796020@163.com (X.S.); 15856589663@163.com (C.P.); 15091422674@163.com (Z.P.); dafu484@whut.edu.cn (D.Z.); hkfy8789@whut.edu.cn (K.H.); 294599@whut.edu.cn (J.Q.); 15251300417@163.com (X.H.); 2 School of Mechanical and Electrical Engineering, Wuhan University of Technology, Wuhan 430070, China; 19870978239@163.com

**Keywords:** nitrate reduction, PtBi alloy, cobalt doping, one-pot synthesis

## Abstract

The electrochemical nitrate reduction reaction (NO_3_RR) represents a promising strategy for wastewater remediation and sustainable ammonia (NH_3_) production. However, its practical application is hindered by low selectivity and competition from the hydrogen evolution reaction (HER). Herein, a series of PtBi-Co_X_ (X = 4.9, 5.3, and 6.1) ternary alloy nanoplates was synthesized via a one-pot method with tunable Co content. Structural characterization indicates that Co incorporation does not significantly alter the hexagonal crystal structure of the PtBi phase. Electrochemical measurements reveal that the NO_3_RR performance varies with PtBi-Co_X_ (X = 4.9, 5.3, 6.1), with PtBi-Co_5.3_ exhibiting the optimal balance of activity and selectivity among the studied samples. At −0.5 V vs. RHE, it achieves a Faradaic efficiency (FE) of 97.75 ± 0.75% and an NH_3_ yield rate of 9.33 ± 0.50 mg h^−1^ mg_cat_^−1^ under the tested conditions. In addition, the catalyst exhibits relatively suppressed HER activity compared to samples with higher Co content, along with good stability. These findings provide useful insights into the design of PtBi-based ternary alloy catalysts for efficient nitrate reduction.

## 1. Introduction

The rapid development of industry and agriculture has led to the discharge of large quantities of nitrogen-containing wastewater [1,2]. Nitrate is the predominant form of nitrogen in such effluents, and its accumulation has caused severe eutrophication in aquatic environments, posing significant threats to ecosystem stability and human health. The electrochemical nitrate reduction reaction (NO_3_RR) offers a promising strategy for both efficient nitrate removal and its conversion into value-added ammonia (NH_3_) under ambient conditions [3,4,5,6], providing a sustainable alternative to the energy-intensive Haber-Bosch process. However, NO_3_RR involves complex multi-electron/proton-coupled processes and multiple reaction intermediates [7,8,9], while the competing hydrogen evolution reaction (HER) further reduces selectivity [10,11,12,13]. Therefore, achieving high Faradaic efficiency (FE) and selectivity strongly depends on the rational design of efficient electrocatalysts.

Platinum (Pt)-based catalysts have been widely used in electrocatalytic reactions, such as the oxygen reduction reaction (ORR) and HER [14,15,16,17,18,19], where they are often alloyed or doped with elements like Ni, Ru, Pd, Rh, and Ir to enhance performance [20]. However, when applied to NO_3_RR, these catalysts typically exhibit low selectivity due to competing side reactions, particularly HER [21,22,23]. To address this issue, researchers have explored the incorporation of elements that promote NO_3_^−^ adsorption, such as Cu, Co, Fe, and Bi [20,24,25]. These elements can effectively suppress HER while improving overall NO_3_RR performance. Among them, Bi facilitates NO_3_^−^ activation and *NO conversion via electronic coupling, which may lower the *NO → *NOH energy barrier and enhance NH_3_ formation [24]. Incorporating Bi into Pt-based catalysts may therefore suppress HER and promote NO_3_^−^ adsorption, as suggested in previous studies. However, PtBi-based catalysts still face challenges related to the limited tunability of active sites and insufficient optimization of catalytic performance [26,27,28,29]. On the other hand, Co has attracted increasing attention due to its strong affinity for oxygen- and nitrogen-containing species [30,31,32,33,34,35,36]. Introducing Co into PtBi-based systems offers a promising strategy to regulate catalytic properties. Nevertheless, despite the extensive studies on binary Pt-based catalysts, ternary PtBiCo systems for NO_3_RR remain largely underexplored, particularly in terms of systematic compositional regulation (e.g., Co content) and its correlation with catalytic performance. More importantly, the correlation between Co content and catalytic performance has not been systematically investigated. In this work, we aim to address this gap by systematically investigating the role of Co incorporation in PtBi-based catalysts. This study provides insights into the effect of Co content on catalytic performance and offers a potential strategy for enhancing both selectivity and activity in NO_3_RR.

Herein, a series of PtBi-Co_X_ ternary alloy nanomaterials was synthesized via a one-pot strategy, in which the Co content can be tuned by adjusting the precursor ratios. The obtained samples exhibit uniform hexagonal nanoplate morphology and well-defined crystal structures. By systematically varying the Co content, the relationship between composition and catalytic performance for NO_3_RR is investigated. Electrochemical results indicate that appropriate Co incorporation can effectively improve catalytic performance. Among the samples, PtBi-Co_5.3_ achieves a high FE of 97.75 ± 0.75% and an NH_3_ yield rate of 9.33 ± 0.50 mg h^−1^ mg_cat_^−1^ at −0.5 V vs. RHE, along with good stability. In addition, the catalyst maintains high activity and selectivity over a wide range of nitrate concentrations, indicating its potential for practical applications. This work provides a systematic study of ternary PtBi-Co_X_ catalysts and highlights the role of compositional tuning in optimizing catalytic performance, offering a feasible strategy for the design of efficient NO_3_RR electrocatalysts.

## 2. Materials and Methods

### 2.1. Materials

Platinum (II) acetylacetonate (Pt(acac)_2_, Aladdin, Shanghai, China, 97%), Bismuth (III) acetate (C_6_H_9_BiO_6_, Aladdin, Shanghai, China, ≥99.9% metals basis), Cobalt (II) acetylacetonate (C_15_H_21_CoO_6_, Aladdin, Shanghai, China, ≥98%), Oleylamine (C_18_H_37_N, Aladdin, Shanghai, China, 80–90%), 1-Octadecene (C_18_H_36_, Aladdin, Shanghai, China, >90.0% GC), Hexadecyl trimethyl ammonium bromide (CTAB, C_19_H_42_BrN, Aladdin, Shanghai, China, ≥99%), Sodium citrate (C_6_H_5_O_7_Na_3_, Aladdin, Shanghai, China, 98%), L-Ascorbic acid (C_6_H_8_O_6_, Sinopharm, Shanghai, China, AR), Ethanol absolute (C_2_H_6_O, Sinopharm, Shanghai, China, AR), Cyclohexane (C_6_H_12_, Sinopharm, Shanghai, China, AR), Ammonium sulfate ((NH_4_)_2_SO_4_, Sinopharm, Shanghai, China, AR), Sodium nitrite (NaNO_2_, Sinopharm, Shanghai, China, AR), Sodium hydroxide (NaOH, Sinopharm, Shanghai, China, AR), Sodium nitrate (NaNO_3_, Sinopharm, Shanghai, China, AR), Phosphoric acid (H_3_PO_4_, Sinopharm, Shanghai, China, AR), Nafion perfluorinated resin solution (5 wt.%, Macklin, Shanghai, China), Salicylic acid (C_7_H_6_O_3_, Macklin, Shanghai, China, 99.5%), Sodium hypochlorite solution (NaClO, Macklin, Shanghai, China, 0.1 mol/L), and N-(1-Naphthyl) ethylenediamine dihydrochloride (C_12_H_14_N_2_·2HCl, Macklin, Shanghai, China, 98%), Sodium nitroprusside (C_5_FeN_6_Na_2_O·2H_2_O, Yuanye, Shanghai, China, 98.5%) and Sulfanilamide (C_6_H_8_N_2_O_2_S, Yuanye, Shanghai, China, 99.8%). Deionized water (Ulupure, Chengdu, China, 18.2 MΩ cm^−1^) was utilized in all experiments.

### 2.2. Methods

A one-pot method was employed for the synthesis of PtBi-Co_X_ alloys, where X denotes the atomic percentage of Co in the material. The detailed experimental procedure is as follows: platinum acetylacetonate (10 mg), bismuth acetate (10 mg), varying amounts of cobalt acetylacetonate (3, 6, and 9 mg), ascorbic acid (40 mg), and cetyltrimethylammonium bromide (CTAB, 200 mg) were added to a reaction flask. Subsequently, oleylamine (5 mL) and 1-octadecene (5 mL) were introduced as a mixed solvent. The reaction mixture was then heated in an oil bath preheated to 180 °C and maintained under magnetic stirring at 600 rpm for 5 h. After completion of the reaction, the solution turned dark reddish-brown with the formation of black precipitates. The obtained products were purified by repeated washing with a mixture of cyclohexane and anhydrous ethanol (*v*/*v* = 1:1), followed by drying at 60 °C to obtain pure PtBi-Co_X_ alloy powders.

### 2.3. Characterization

The crystal structure of the samples was analyzed by X-ray diffraction (XRD) using a Rigaku Ultima IV diffractometer (Tokyo, Japan) using Cu-Kα radiation (λ = 1.54 Å) at a scanning rate of 2° min^−1^. The morphology and elemental distribution of the samples were characterized by transmission electron microscopy (TEM) using a JEOL JEM-1400 Plus microscope (Tokyo, Japan) and a Talos F200S field-emission transmission electron microscope (Thermo Fisher Scientific, Waltham, MA, USA). The chemical states of the elements were analyzed by X-ray photoelectron spectroscopy (XPS) on a Thermo Fisher K-Alpha spectrometer (Waltham, MA, USA) with an Al Kα X-ray source (E = 1486.68 eV). The reaction products were quantitatively analyzed using a Shimadzu UV-2550 UV-visible spectrophotometer (Kyoto, Japan).

### 2.4. Electrochemical Measurements

All electrochemical measurements were carried out on a CS310H electrochemical workstation using a standard three-electrode configuration in an H-type electrochemical cell. A platinum plate (1 cm × 1.5 cm) served as the counter electrode, and a Hg/HgO electrode (immersed in 1 M KOH) was used as the reference electrode. The working electrode consisted of catalyst-loaded carbon paper fixed with a platinum clip, with a geometric area of 1 cm^2^. The working electrode was prepared as follows: 1 mg of catalyst was dispersed in 960 μL of an ethanol-water mixed solvent (ethanol/deionized water = 3:1, v/v), followed by the addition of 40 μL Nafion solution. The mixture was ultrasonicated at low temperature for 30 min to form a homogeneous catalyst ink. Subsequently, 100 μL of the ink was uniformly drop-cast onto the carbon paper, resulting in a catalyst loading of 0.2 mg cm^−2^.

Prior to measurements, the working electrode was activated in Ar-saturated 1 M NaOH by cyclic voltammetry over a potential range of 0–1.2 V vs. RHE at a scan rate of 100 mV s^−1^ for 30 cycles, to clean and stabilize the catalyst surface and achieve a steady electrochemical state. The anodic compartment was filled with 30 mL of 1 M NaOH, while the cathodic compartment contained 30 mL of a mixed electrolyte consisting of 1 M NaOH and 0.5 M NaNO_3_. The two compartments were separated by a Nafion 117 membrane. Prior to testing, the catholyte was continuously purged with Ar for at least 30 min to remove dissolved oxygen and nitrogen.

Chronoamperometric (CA) measurements were conducted at a series of applied potentials, with continuous magnetic stirring at 500 rpm during electrolysis. Linear sweep voltammetry (LSV) was performed over a potential range of −0.8 to 0 V vs. RHE at a scan rate of 10 mV s^−1^. All potentials reported in this work are presented without iR compensation to reflect the raw experimental data and avoid uncertainties associated with resistance estimation [37]. The performance comparison is primarily based on relative activity among samples with different Co contents under identical conditions. The catalytic performance was evaluated using both geometric and mass-normalized current densities, based on the geometric area of the working electrode and the catalyst loading. All measured potentials were converted to the reversible hydrogen electrode (RHE) scale according to Equation (1), where E_Hg/HgO_ is the measured potential versus the Hg/HgO (1 M KOH) reference electrode, and E_RHE_ is the converted potential versus RHE. This conversion ensures a consistent potential reference for comparison with literature data.

(1) $$E_{RHE}=E_{Hg/HgO}+0.0591\times pH+0.098$$

The electrochemically active surface area (ECSA) was estimated from the double-layer capacitance (C_dl_). Cyclic voltammetry was performed within a non-Faradaic potential window at various scan rates ranging from 20 to 120 mV s^−1^. The charging current density (I_c_) at a selected potential exhibited a linear relationship with the scan rate (v), as described in Equation (2). The slope of the linear fit corresponds to the C_dl_. The ECSA was then calculated using Equation (3), where Cs represents the specific capacitance of the catalyst. A constant Cs value of 40 μF cm^−2^ was assumed (within the typical range of 20–60 μF cm^−2^), which has been widely adopted in the literature for materials with similar surface chemistry [37,38]. It should be noted that the estimation of intrinsic activity based on ECSA is subject to uncertainty due to the assumption of a constant specific capacitance. Therefore, in this work, the comparison is primarily based on the relative performance under identical conditions.

(2) $$I_{c}=vC_{dl}$$

(3) $$ECSA=C_{dl}/C_{s}$$

### 2.5. Concentration Detection

The concentration of NH_3_ in the electrolyte was determined using the indophenol blue colorimetric method [39,40,41]. Three color-developing reagents were first prepared as follows: reagent A consisted of a 1 M NaOH solution containing 5 wt% salicylic acid and 5 wt% sodium citrate; reagent B was a 2 M NaOH solution containing 0.05 M NaClO; and reagent C was a 1 wt% sodium nitroprusside solution. For sample analysis, 2 mL of appropriately diluted electrolyte was taken, followed by the sequential addition of 2 mL of reagent A, 1 mL of reagent B, and 0.2 mL of reagent C. The mixture was thoroughly mixed and allowed to stand for 2 h to complete the color development reaction. Subsequently, the absorbance at 655 nm was measured using a UV-vis spectrophotometer.

The concentration of nitrite in the electrolyte was determined using the N-(1-naphthyl) ethylenediamine dihydrochloride colorimetric method [39,40,41]. The color-developing reagent was prepared by dissolving 4 g of sulfanilamide and 0.2 g of N-(1-naphthyl) ethylenediamine dihydrochloride in a mixture of 50 mL of deionized water and 10 mL of phosphoric acid. For sample analysis, 5 mL of appropriately diluted electrolyte was taken, followed by the addition of 0.1 mL of the color-developing reagent. The mixture was thoroughly mixed and allowed to react for 20 min for color development. Subsequently, the absorbance at 540 nm was measured using a UV-vis spectrophotometer.

## 3. Results and Discussion

**Figure 1:**
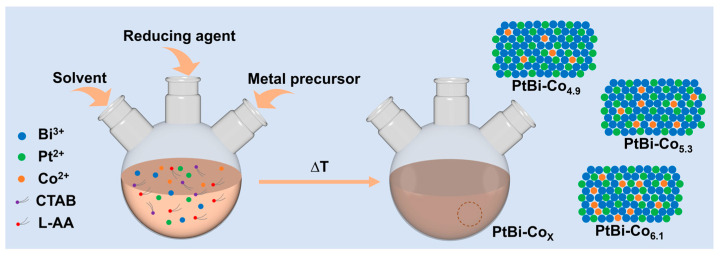
Schematic illustration of the synthesis strategy for PtBi-Co_X_ (X = 4.9, 5.3, 6.1) alloys.

Figure 1 schematically illustrates the synthesis strategy of the PtBi-Co_X_ alloys. The synthesis was carried out in a mixed solvent system consisting of oleylamine and 1-octadecene. Ternary alloys were obtained via the co-reducing platinum acetylacetonate, bismuth acetate, and cobalt acetylacetonate precursors. In this system, oleylamine acts as the solvent, coordinating agent, and mild reducing agent, while ascorbic acid serves as the primary reducing agent. Cetyltrimethylammonium bromide (CTAB) functions as a surfactant, facilitating the uniform dispersion of metal species through coordination with the precursors. Magnetic stirring was initially applied at room temperature to ensure complete dissolution of the precursors and homogeneous mixing, thereby promoting complexation. This step is important for achieving uniform nucleation and growth during the subsequent high-temperature reduction process.

To further investigate the effect of reaction time on particle growth, a series of PtBiCo catalysts was synthesized with a fixed cobalt precursor amount of 3 mg and reaction times of 1, 3, 5, and 7 h (Figure S1). At 180 °C, different metal ions may undergo distinct reduction processes. Previous studies suggest that Pt^2+^ and Bi^3+^ are reduced earlier, possibly leading to the formation of Pt-Bi-rich nuclei [26,27,28,29]. In contrast, Co^2+^, which exhibits relatively slower reduction kinetics, may be incorporated into the preformed Pt-Bi lattice during subsequent crystal growth. It should be noted that the precise reduction sequence and incorporation mechanism remain unclear and require further investigation. TEM images obtained at reaction times of 1, 3, 5, and 7 h show similar particle sizes, indicating that most particle growth occurs within the first hour. Thereafter, the particle size remains relatively stable, with no significant changes observed even after prolonged reaction times. This suggests that particle growth is largely completed within the initial stage, followed by a stabilization process. Nevertheless, a reaction time of 5 h was selected based on previous reports to ensure good uniformity and an appropriate particle size for subsequent electrochemical measurements [29].

**Figure 2:**
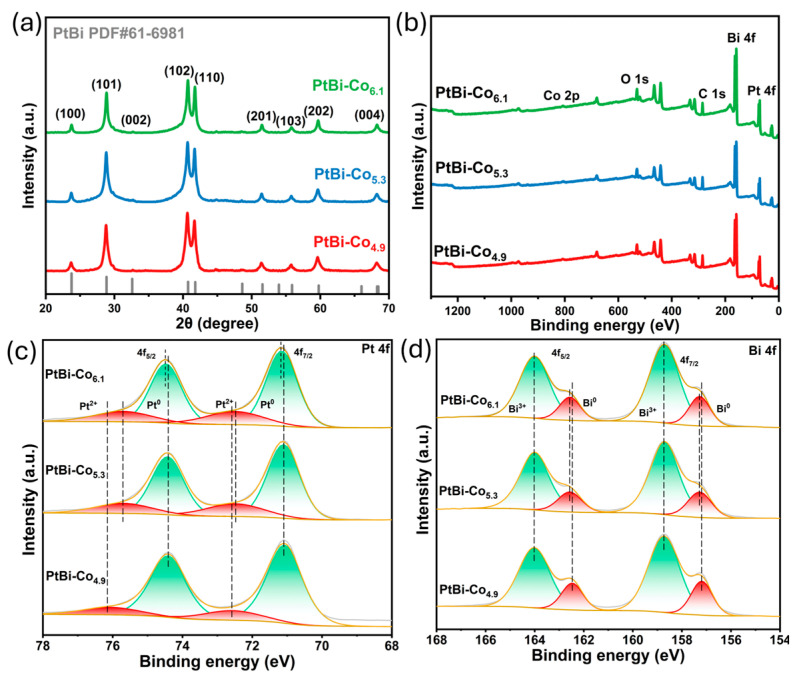
(**a**) X-ray diffraction (XRD) patterns, (**b**) X-ray photoelectron spectroscopy (XPS) survey spectra, high-resolution (**c**) Pt 4f and (**d**) Bi 4f spectra of the PtBi-Co_X_ (X = 4.9, 5.3, 6.1) samples.

By varying the amount of cobalt acetylacetonate precursor, the Co content in the alloy increases accordingly, enabling the synthesis of a series of PtBi-Co_X_ (X = 4.9, 5.3, 6.1) alloys with different compositions. The value of X in PtBi-Co_X_ was determined based on compositional analysis obtained from XPS (Table S2). Figure 2a shows the XRD patterns of the samples. The diffraction peaks can be well indexed to a hexagonal phase (space group P6_3_/mmc) according to the standard PDF card (PDF#61-6981), with no detectable impurity phases, indicating good crystallinity. Enlarged XRD patterns (Figure S2, Table S1) reveal that the (101), (102), and (110) peaks gradually shift to higher angles as the Co content increases. This shift corresponds to a decrease in interplanar spacing and may suggest lattice contraction associated with Co incorporation. Such changes may be associated with structural variations upon Co incorporation, although more direct evidence is required to confirm this interpretation. The XPS survey spectra (Figure 2b) confirm the presence of Pt, Bi, and Co in the samples. However, due to the relatively low Co content, the Co 2p signal is too weak to allow reliable acquisition of high-resolution spectra, making detailed analysis of the Co chemical state infeasible. As shown in Figure 2c,d, Pt predominantly exists in the metallic state, while Bi is mainly present in an oxidized form. The binding energy of Pt^2+^ shifts toward lower values, suggesting that the electron density of Pt in the PtBi-Co_X_ system may increase. Meanwhile, the binding energy of Bi^0^ shifts toward higher values, indicating that the electron-deficient state of Bi atoms may be partially alleviated. Previous studies have suggested that electron transfer from Bi to Pt can occur in similar multimetallic systems [29]. Therefore, with increasing Co content, the variations in Pt^2+^ and Bi^0^ can serve as indirect evidence, suggesting that the incorporation of Co may contribute to the observed electronic modulation of Pt [42]. It should be emphasized that these interpretations are based on indirect evidence, and the precise electronic interactions and atomic-scale structural features will require further investigation using more advanced characterization techniques.

**Figure 3:**
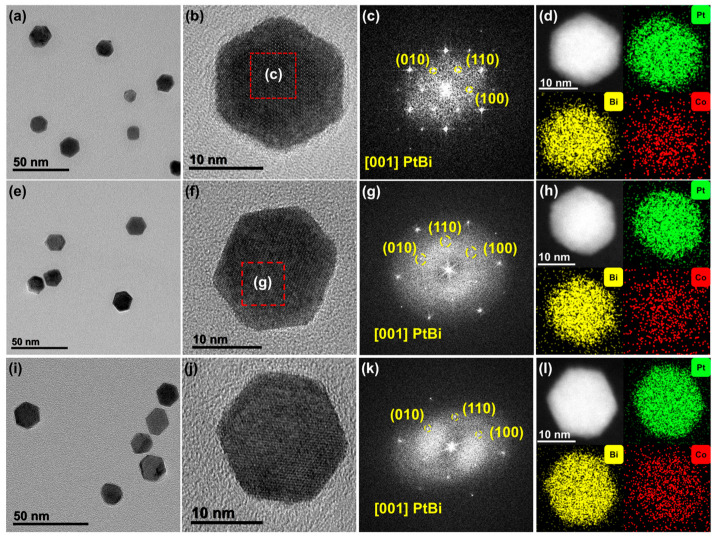
(**a**,**b**,**e**,**f**,**i**,**j**) High-resolution transmission electron microscopy (HRTEM) images, (**c**,**g**,**k**) fast Fourier transform (FFT) patterns, and (**d**,**h**,**l**) energy-dispersive X-ray spectroscopy (EDS) elemental mappings of PtBi-Co_X_ (X = 4.9, 5.3, 6.1) alloy nanoplates.

The morphology and elemental distribution of the samples were characterized by high-resolution transmission electron microscopy (HRTEM) and energy-dispersive X-ray spectroscopy (EDS) (Figure 3). Low-magnification HRTEM images of the PtBi-Co_X_ alloys (Figure 3a,e,i) show that the nanoparticles exhibit a well-defined hexagonal nanoplate morphology, with an average edge length of ~10 nm. The morphology remains largely unchanged with varying Co content, suggesting that the introduction of Co does not significantly affect the overall growth behavior of the PtBi-based alloy [29]. These results also imply good synthetic stability and reproducibility. High-resolution TEM images (Figure 3b,f,j) reveal clear lattice fringes, indicating good crystallinity. The corresponding fast Fourier transform (FFT) patterns (Figure 3c,g,k) exhibit hexagonal symmetry, with diffraction spots indexed to the (110), (100), and (010) planes, consistent with the PtBi phase viewed along the [001] direction. This agrees with the XRD results (Figure 2a and Figure S2). EDS elemental mapping (Figure 3d,h,l) shows that Pt and Bi are relatively uniformly distributed across the nanoplates, without obvious large-scale elemental segregation. The atomic ratios obtained from EDS analysis are consistent with those derived from XPS, supporting the reliability of the compositional analysis (Tables S2 and S3). Taken together, the uniform elemental distribution observed by EDS, along with the absence of impurity-phase peaks and the systematic shift in diffraction peaks in XRD, suggests that Co is likely incorporated into the PtBi lattice to form an alloyed structure at the detectable scale. However, atomic-scale homogeneity and the precise distribution of Co cannot be conclusively determined based on the current characterization results.

**Figure 4:**
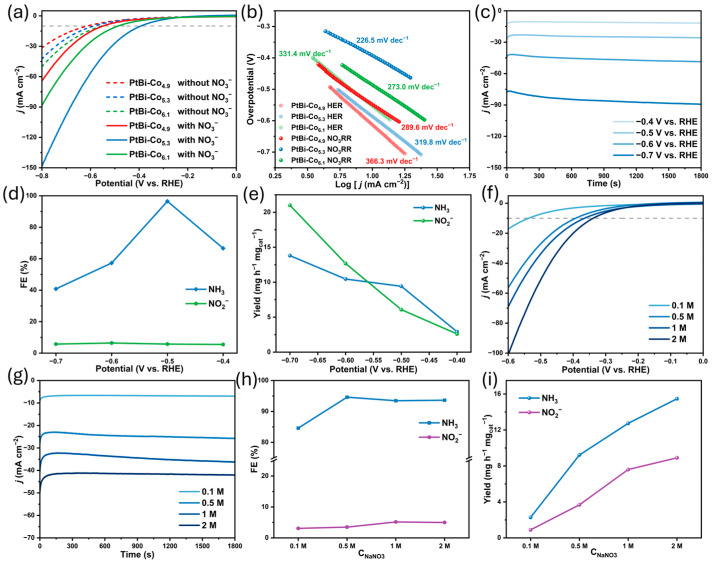
Electrocatalytic nitrate reduction reaction (NO_3_RR) performance of PtBi-Co_X_ (X = 4.9, 5.3, 6.1) alloys. (**a**) Linear sweep voltammetry (LSV) curves and (**b**) Tafel plots of PtBi-Co_X_. (**c**) Chronoamperometric (CA) curves, (**d**) Faradaic efficiency (FE) and (**e**) NH_3_ yield rate of PtBi-Co_5.3_ at various potentials. (**f**) LSV curves of PtBi-Co_5.3_ at varying NO_3_^−^ concentrations. (**g**) CA curves, (**h**) FE and (**i**) NH_3_ yield rate of PtBi-Co_5.3_ at different NO_3_^−^ concentrations.

Based on the above results, the electrocatalyticNO_3_RR performance of PtBi-Co_X_ (X = 4.9, 5.3, 6.1) alloys was systematically evaluated in 1 M NaOH with 0.5 M NaNO_3_. As shown in theLSV curves (Figure 4a), the NO_3_RR activity varies with Co content, with PtBi-Co_5.3_ exhibiting the highest activity among the samples. A non-monotonic dependence on Co content is observed, possibly related to changes induced by Co incorporation. All PtBi-Co_X_ catalysts exhibit relatively suppressed HER activity, as indicated by their relatively large overpotentials for HER. As shown in Figure S3, however, the potentials required to reach current densities of 10 and 30 mA cm^−2^ decrease with increasing Co content, suggesting a gradual enhancement of HER activity. Among the samples, PtBi-Co_6.1_ shows the most pronounced HER behavior. These results indicate that excessive Co incorporation may promote HER under nitrate-free conditions, which could be detrimental to NO_3_RR selectivity. To further evaluate reaction kinetics, Tafel plots were derived from the LSV curves (Figure 4b). The Tafel slopes suggest that PtBi-Co_5.3_ may exhibit faster nitrate reduction kinetics compared with PtBi-Co_4.9_ and PtBi-Co_6.1_, consistent with its higher activity. The electrochemically active surface area was estimated from C_dl_ measurements (Figure S4). PtBi-Co_5.3_ exhibits a C_dl_ value of 0.352 mF cm^−2^, which lies between those of PtBi-Co_4.9_ (0.332 mF cm^−2^) and PtBi-Co_6.1_ (0.374 mF cm^−2^). Given the similar crystal structures and relatively low Co content, these small differences suggest that the observed activity trends are unlikely to be dominated by surface area effects [38]. Therefore, the catalytic performance comparison in this work is based on relative activity under identical experimental conditions rather than a strict evaluation of intrinsic activity. Overall, PtBi-Co_5.3_ demonstrates the most favorable performance among the studied samples under the tested conditions.

CA tests were performed on the PtBi-Co_5.3_ catalyst in 1 M NaOH with 0.5 M NaNO_3_ at various applied potentials (Figure 4c). NH_3_ production was quantified using the indophenol blue colorimetric method, while NO_2_^−^ intermediates were measured using the N-(1-naphthyl)ethylenediamine dihydrochloride method (Figure S5) [39,40,41]. Based on these measurements, the FE and NH_3_ production rate of PtBi-Co_5.3_ at different potentials were determined (Figure 4d,e). The results show that PtBi-Co_5.3_ exhibits high selectivity for NH_3_ across the tested potential range. At −0.5 V vs. RHE, the FE for NH_3_ reached 96.48%, with an NH_3_ production rate of 9.41 mg h^−1^ mg_cat_^−1^. Table S4 summarizes the performance of reported noble-metal-based catalysts [12,23,43,44,45,46]. Comparison indicates that Pt-based catalysts generally exhibit higher yields in theNO_3_RR, confirming Pt as a key metal for efficient NO_3_RR. Notably, the PtBi-Co_5.3_ developed in this study demonstrates enhanced performance under moderate Pt loadings, achieving comparable or slightly higher FE at lower overpotentials than many reported Pt-based catalysts, suggesting improved suppression of the competing HER. However, the optimization of our PtBi-Co_X_ catalyst is limited by the allowable Co content, which constrains the maximal achievable activity and selectivity. Future catalyst design could further incorporate interfacial engineering strategies to address the complex reaction pathways of NO_3_RR, which may lead to improved efficiency and selectivity [20].

To assess the concentration adaptability of PtBi-Co_5.3_, the electrocatalytic performance was further evaluated over a range of nitrate concentrations from 0.1 M to 2 M. As shown in Figure 4f, the current density in the LSV curves increases with nitrate concentration, suggesting an enhancement in reaction kinetics. CA tests conducted at varying NO_3_^−^ concentrations (Figure 4g,h,i) show that PtBi-Co_5.3_ maintains relatively high FE and NH_3_ production across the tested range. Even at a low concentration of 0.1 M NO_3_^−^, the FE reached 84.61%, indicating that the catalyst remains active under varying nitrate loadings. These observations provide valuable insights into the catalyst’s performance under different nitrate concentrations. However, at higher nitrate concentrations (up to 2 M), mass transport effects, including diffusion limitations and potential local pH variations, may influence the observed catalytic behavior. A more detailed investigation would be required to fully clarify these effects. It should be noted that a complete nitrogen balance was not established in this study. The product analysis focused primarily on NH_3_ quantification, while only the major byproduct (NO_2_^−^) was detected and quantified due to experimental limitations. As a result, other possible nitrogen-containing products, such as N_2_, NO, N_2_O, or hydroxylamine (NH_2_OH), as reported in similar NO_3_RR systems, may not have been fully accounted for. A fully quantitative nitrogen balance is beyond the scope of the present study, as comprehensive detection of all possible nitrogen-containing products, particularly gaseous species, would typically require additional dedicated analytical techniques. The presence of these undetected products may lead to a certain degree of overestimation of the reported NH_3_ selectivity, particularly under conditions where competing pathways are more pronounced, such as at more negative potentials or higher current densities. Therefore, the selectivity values presented here should be interpreted as a relative comparison under the tested conditions rather than a comprehensive evaluation of the full nitrogen product distribution.

**Figure 5:**
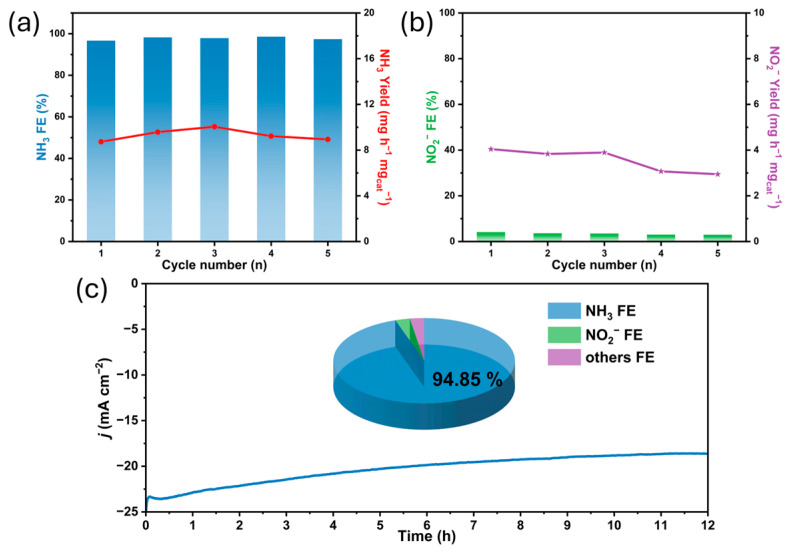
(**a**,**b**) Cyclic stability test of PtBi-Co_5.3_. (**c**) Long-term CA stability test of PtBi-Co_5.3_ over 12 h.

Additionally, the electrocatalytic stability of the PtBi-Co_5.3_ alloy was evaluated. The catalyst underwent five consecutive cycling tests (Figure 5a,b), during which the FE for NH_3_ production was maintained at 97.75 ± 0.75%, with an NH_3_ yield rate of 9.33 ± 0.50 mg h^−1^ mg_cat_^−1^. The small variation in these values indicates good reproducibility and stability under the tested conditions, with no significant performance decay over repeated cycles. Long-term stability was further investigated via a 12 h CA test. As shown in Figure 5c, a slight decrease in current density occurred at the initial stage of electrolysis, followed by gradual stabilization (Figure S6). At the end of the test, the FE for NH_3_ remained at 94.85%, indicating sustained catalytic activity over time. To examine structural stability, the catalyst was collected from carbon paper after electrolysis and characterized by TEM (Figure S7). The results show that the hexagonal nanoplate morphology is largely preserved, suggesting that the catalyst maintains its structural integrity during operation. Control experiments were conducted to exclude potential contributions from other sources (Figure S8). The bare carbon paper electrode produced only a negligible amount of NH_3_ (0.407 mg h^−1^ mg_cat_^−1^) at −0.5 V vs. RHE. Almost no NH_3_ was detected in nitrate-free electrolyte or under open-circuit potential (OCP) conditions, confirming that the detected NH_3_ primarily originates from the electrochemical reduction of NO_3_^−^ under the present experimental conditions. Although Pt-based catalysts exhibit high activity, their high cost and limited availability may hinder large-scale applications. Incorporation of Co partially reduces the reliance on noble metals, and the formation of Pt-Bi-based alloys further decreases the relative Pt usage. Such alloying strategies may provide a potential pathway to balance catalytic performance and economic feasibility.

## 4. Conclusions

In summary, a series of PtBi-Co_X_ ternary alloy nanoplates was synthesized via a one-pot co-reduction strategy. Structural characterization suggests that the incorporation of Co largely preserves the hexagonal crystal structure of the PtBi phase. Combined XRD, TEM, and XPS analyses suggest the incorporation of Co into the PtBi lattice at the detectable scale, although the detailed atomic arrangement requires further investigation. Electrochemical measurements show that the catalytic performance toward NO_3_RR varies with PtBi-Co_X_ (X = 4.9, 5.3, 6.1), with PtBi-Co_5.3_ exhibiting the most favorable activity and selectivity among the studied samples. At −0.5 V vs. RHE, PtBi-Co_5.3_ achieves a FE of 97.75 ± 0.75% and an NH_3_ yield rate of 9.33 ± 0.50 mg h^−1^ mg_cat_^−1^ under the tested conditions. The catalyst also shows relatively lower HER activity compared with higher Co-content samples and maintains stable performance over a range of nitrate concentrations (0.1–2 M). In addition, PtBi-Co_5.3_ demonstrates good stability under the tested conditions, retaining high FE during repeated cycling and prolonged electrolysis. It should be noted that a complete nitrogen balance was not established, and reaction pathways as well as intermediate species were not directly investigated. Therefore, the discussion is based on observed experimental trends, without drawing definitive mechanistic conclusions. Overall, this work provides insight into the influence of Co incorporation on the catalytic performance of PtBi-based materials and may offer useful guidance for the design of electrocatalysts for NO_3_RR under similar conditions.

## Data Availability

The original contributions presented in this study are included in the article/Supplementary Materials. Further inquiries can be directed to the corresponding author.

## Supplementary Materials

The following supporting information can be downloaded at: https://www.mdpi.com/article/10.3390/ma19101953/s1, Figure S1: Synthesis of PtBiCo catalysts with varying reaction times; Figure S2: Enlarged XRD patterns of the PtBi-Co_X_ (X = 4.9, 5.3, 6.1) samples; Figure S3: Overpotentials of PtBi-Co_X_ (X = 4.9, 5.3, 6.1) catalysts at 10 and 30 mA cm^−2^; Figure S4: (a–c) CV curves of PtBi-Co_X_ (X = 4.9, 5.3, 6.1) catalysts during NO_3_RR at various scan rates; (d–f) corresponding plots of current density difference versus scan rate for the catalysts; Figure S5: (a,b) Calibration curve for NH_3_ concentration determination using the indophenol blue method. (c,d) Calibration curve for NO_2_^−^ concentration determination using the N-(1-naphthyl) ethylenediamine dihydrochloride method; Figure S6: The yield rate of NH_3_ and NO_2_^−^ after stability test; Figure S7: TEM image of PtBi-Co_5.3_ catalyst after electrolysis; Figure S8: Control experiments for NH_3_ production; Table S1: XRD diffraction peaks and corresponding 2θ value; Table S2: Atomic percentages of elements measured by XPS. Table S3: Atomic percentages of elements measured by EDS; Table S4: Comparison of NO_3_RR performance with literature.
