# Supplementary material for: One-Pot Synthesis of PtBi-CoX Alloys for Electrochemical Nitrate Reduction to Ammonia
Source: Materials (Basel). 2026 May 9;19(10):1953. doi: 10.3390/ma19101953 (PMC13208363; doi:10.3390/ma19101953)
Supplement: Supplementary file 1 [file materials-19-01953-s001.zip › materials-4242140-supplementary.pdf]

Supplementary materials

One-Pot Synthesis of PtBi-CoX Alloys for Electrochemical Nitrate Reduction to Ammonia

Yingfei Liu [1,†], Yuxuan Wang [2,†], Xiyuan Sun [1], Chong Peng [1], Zhe Pang [1], Dafu Zhao [1], Kefeiyang Hu [1], Jiaqian Que [1], Xingbo Huang [1] and Yong Liu [1,*]

1State Key Laboratory of Advanced Technology for Materials Synthesis and Processing, School of Materials Science and Engineering, Wuhan University of Technology, Wuhan 430070, China; liu714961@163.com (Y.L.); 18952796020@163.com (X.S.); 15856589663@163.com (C.P.); 15091422674@163.com (Z.P.); dafu484@whut.edu.cn (D.Z.); hkfy8789@whut.edu.cn (K.H.); 294599@whut.edu.cn (J.Q.); 15251300417@163.com (X.H.)

[2]School of Mechanical and Electrical Engineering, Wuhan University of Technology, Wuhan 430070, China; 19870978239@163.com

*Correspondence: liuyong3873@whut.edu.cn

[†] These authors contributed equally to this work.

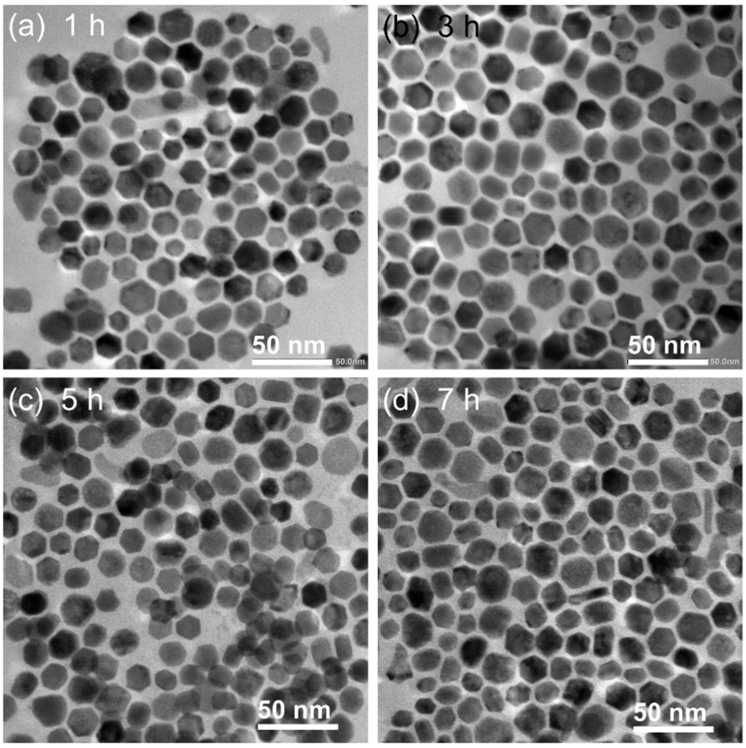

Figure S1. Synthesis of PtBiCo catalysts with varying reaction times.

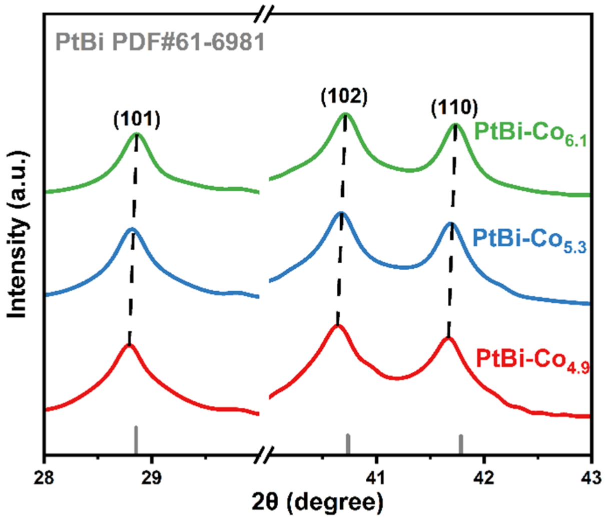

Figure S2. Enlarged XRD patterns of the PtBi-Co$_X$ (X = 4.9, 5.3, 6.1) samples.

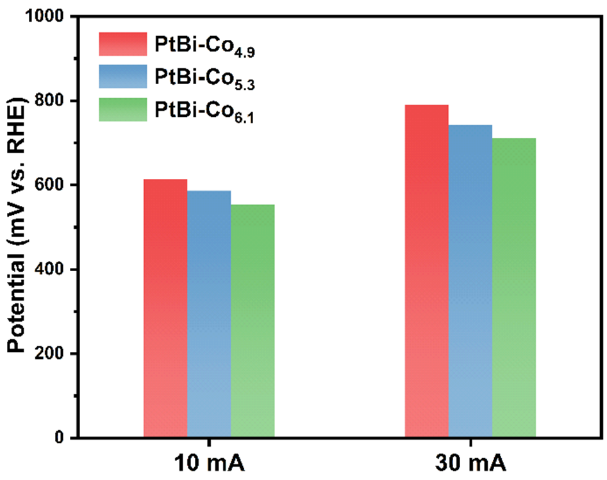

Figure S3. Overpotentials of PtBi-CoX (X = 4.9, 5.3, 6.1) catalysts at 10 and 30 mA cm^{-2}.

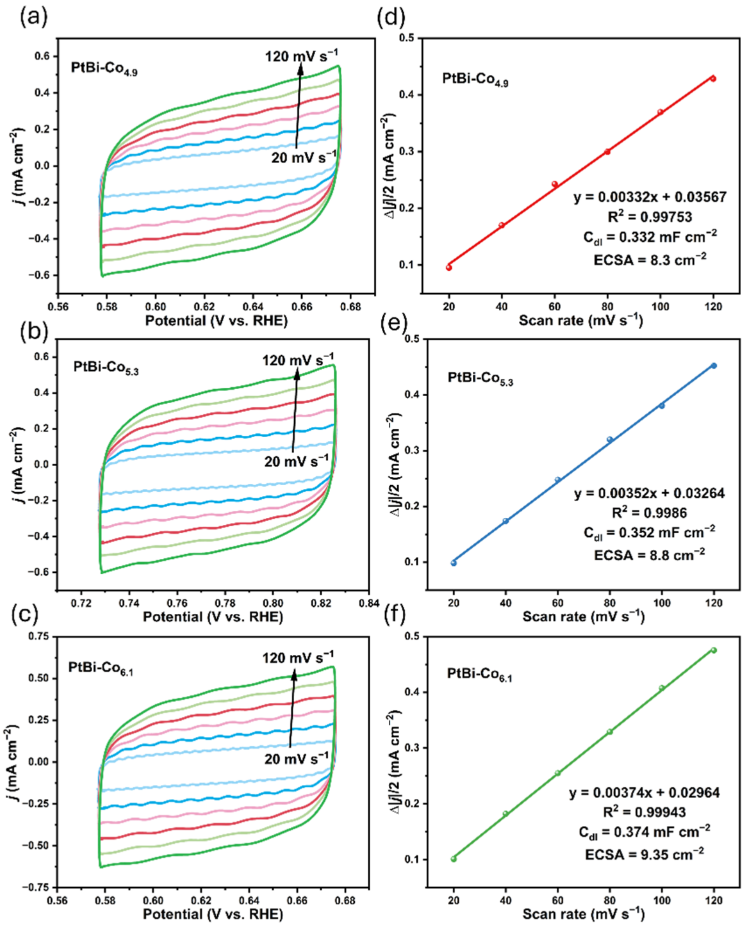

Figure S4. (a-c) CV curves of PtBi-CoX (X = 4.9, 5.3, 6.1) catalysts during NO_3RR at various scan rates; (d-f) corresponding plots of current density difference versus scan rate for the catalysts.

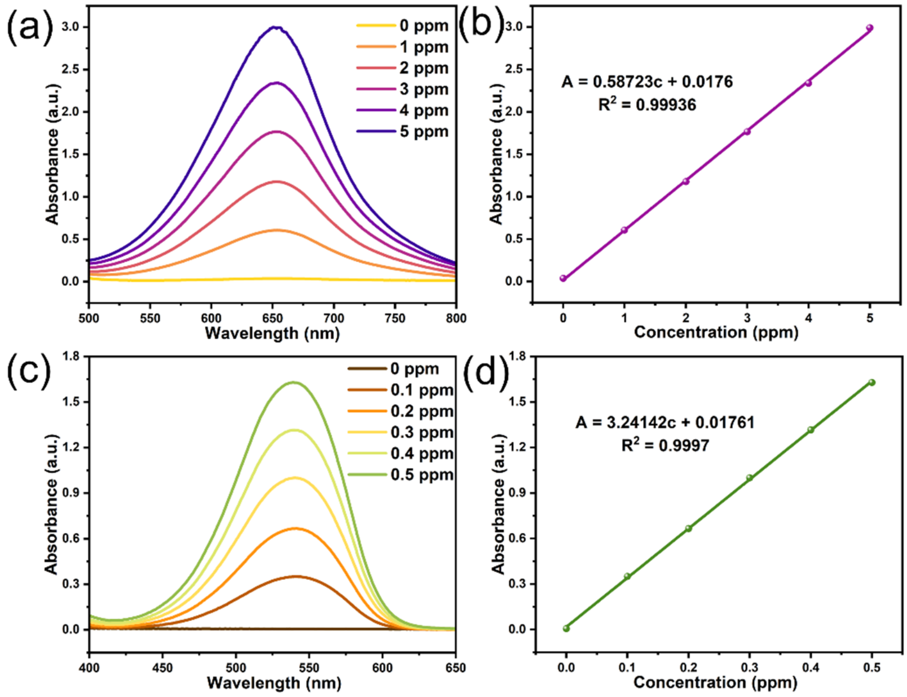

Figure S5. (a-b) Calibration curve for NH_3 concentration determination using the indophenol blue method. (c-d) Calibration curve for NO_2^- concentration determination using the N-(1-naphthyl) ethylenediamine dihydrochloride method.

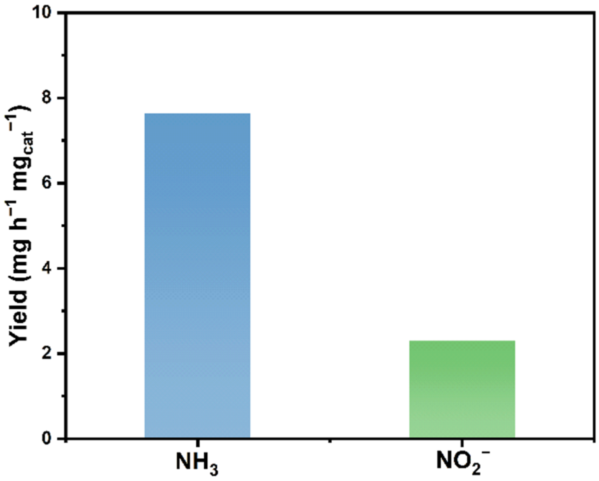

Figure S6. The yield rate of NH_3 and NO_2^- after stability test.

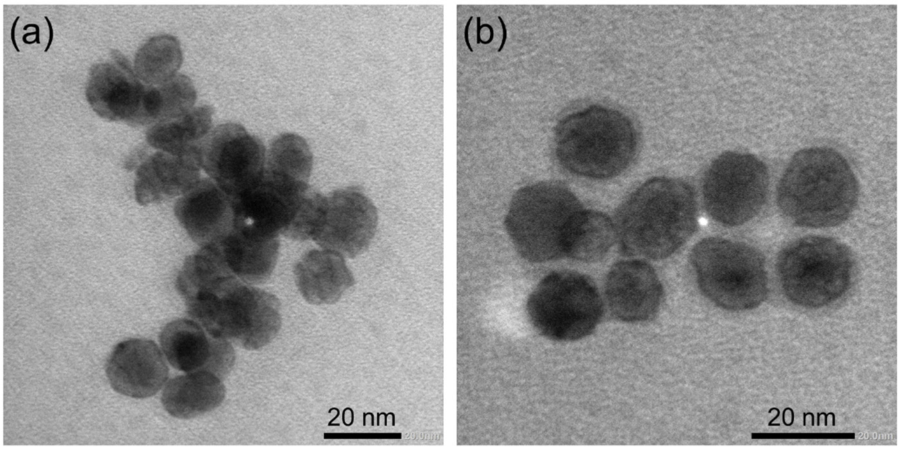

Figure S7. TEM image of PtBi-$Co_{5.3}$ catalyst after electrolysis.

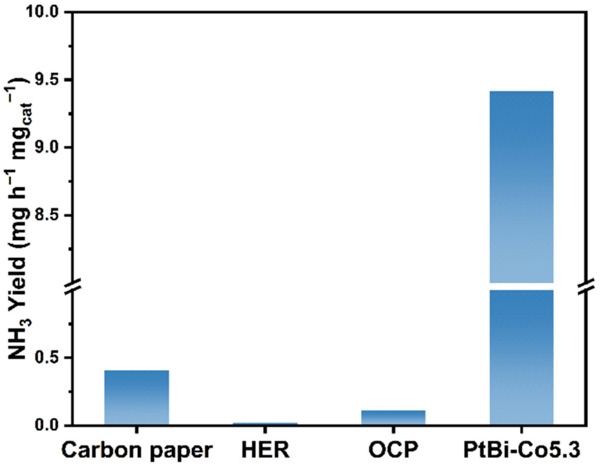

Figure S8. Control experiments for NH_3 production.

Table S1. XRD diffraction peaks and corresponding 2θ value.

	PtBi-$Co_{4.9}$	PtBi-$Co_{5.3}$	PtBi-$Co_{6.1}$
(101)	28.80	28.82	28.86
(102)	40.64	40.68	40.72
(110)	41.66	41.7	41.74

Table S2. Atomic percentages of elements measured by XPS.

Cat.	Pt(%)	Bi(%)	Co(%)
PtBi-$Co_{4.9}$	45.42	49.63	4.95
PtBi-$Co_{5.3}$	44.05	50.65	5.30
PtBi-$Co_{6.1}$	43.78	50.08	6.14

Table S3. Atomic percentages of elements measured by EDS.

Cat.	Pt(%)	Bi(%)	Co(%)
PtBi-$Co_{4.9}$	44.91	50.14	4.95
PtBi-$Co_{5.3}$	43.48	50.84	5.68
PtBi-$Co_{6.1}$	42.11	51.44	6.44

Table S4. Comparison of NO_3RR performance with literature.

Catalyst	Faradaic efficiency	The optimal potential for FE	NH_3 yield rate	Reference
**PtBi-$Co_{5.3}$**	**98.5%**	**−0.5 V vs. RHE**	**9.80 mg h^{-1} mg_{cat}^{-1}**	**This work**
PdCu SAA	97.1%	−0.6 V vs. RHE	15.4 μmol cm^{-2} h^{-1}	[42]
Pt-BCN	91.79%	−1 V vs .RHE	12.71 mg h^{-1} mg_{cat}^{-1}	[23]
Ru-$Ni(OH)_2$	99.7%	0 V vs. RHE	≈ 5 mg cm^{-2} h^{-1}	[43]
Ru-Fe_2O_3	72.8%	−0.9 V vs. RHE	329 μmol cm^{-2} h^{-1}	[44]
$Pt_{0.8}Fe_{0.2}Co_{0.2}Ni_{0.2}Cu_{0.2}$	98.8%	−0.3 V vs. RHE	≈ 9 mg cm^{-2} h^{-1}	[45]
Au_1Cu SAA	98.7%	−0.2 V vs. RHE	555 μg cm^{-2} h^{-1}	[12]
